# From imperial examination to digital soloing: why Generation Z resist mandatory cooperation in Chinese higher education

**DOI:** 10.3389/fpsyg.2026.1800462

**Published:** 2026-04-09

**Authors:** Guosheng Xu

**Affiliations:** Applied Translation Studies Center, Shandong Huayu University of Technology, Dezhou, Shandong, China

**Keywords:** cooperative project-based learning, Generation Z, imperial examination system, performative collaboration, strategic soloing

## Abstract

**Introduction:**

Project-based learning (PBL) is promoted in Chinese higher education, but Generation Z students often display “strategic soloing” resisting mandatory cooperation despite policy expectations. Existing studies emphasize the pedagogical benefits of PBL but pay limited attention to how historical legacies, surveillance technologies, and digital-era collaboration norms jointly shape learners’ motivation in Chinese contexts.

**Methods:**

This 4-month educational ethnography examined 150 first-year students from four English classes in one Chinese university, among whom 20 were interviewed. Data included 40 h of classroom observation, 40 video-recorded sessions, 40 SuperStar log traces, and WeChat-based documents. Data were analyzed through reflective thematic analysis and micro-interaction coding.

**Results:**

The findings reveal three intertwined mechanisms. First, imperial-examination formalism persists in assessment practices: 30% of group-report grading weight was allocated to formatting, whereas only 10% assessed collaboration, leading students to prioritize procedural compliance. Second, a Foucauldian surveillance triad involving teachers, cameras, and peer assessments generated performative collaboration; video data showed discussion volume rising sharply only during teacher proximity. Third, digital-native collaboration ethics, shaped by gameplay metrics such as DPS (damage per second) statistics, conflicted with high-context classroom norms; 75% of interviewees reported higher trust and efficiency in virtual guilds than in classroom groups.

**Discussion:**

The study suggests that PBL assessment should be recalibrated toward transparent contribution metrics and reduced surveillance-based performativity. Theoretically, it proposes the “imperial examination–meta-cosmos” framework to explain how cultural legacies and digital cognition together produce resistance to institutionalized cooperation.

## 1 Introduction

In recent years, Chinese higher education has increasingly adopted project-based learning (PBL) as part of broader reforms to cultivate students’ comprehensive competencies (Eryong and Li, 2021; Shin, 2018). This shift toward project-based collaborative learning is driven by Chinese higher education reforms. Under the New Liberal Arts initiative^1^ and related curriculum reforms, Chinese universities began to redesign courses around interdisciplinary projects, collaboration, and employment skills (Liu et al., 2025). PBL is now institutionalized to strengthen innovation and employability (Domingues et al., 2024; Jackson, 2016), particularly in Chinese EFL learning, where it is formally embedded in teaching guidelines and assessment systems (Sun and Zhu, 2023).

In principle, PBL emphasizes collaboration, knowledge co-construction, and authentic engagement (Johnson and Mislin, 2011). Yet, in Chinese classrooms, particularly in English language courses, many Generation Z students display a paradoxical preference for independent work, even when tasks are designed to be completed in a collaborative manner. Thus a gap is created between policy expectations and students’ actual learning behaviors. Collaborative project work is institutionalized, and students often adopt adaptive strategies based upon how performance is assessed, how cooperation is monitored by teachers and platforms, and how much control they can have over the outcome (Zhong et al., 2025). This behavior shows a mismatch between university requirements for group work and students’ practical strategies.

An important historical influence is the imperial examination system^2^, which for over a millennium shaped China’s educational ethos. As Elman (2013) notes, the system institutionalized formalism, privileging compliance with rigid formats (e.g., vermilion ink transcription^3^) over substantive creativity. This legacy persists in modern Chinese assessment structures, where groupwork is often evaluated by procedural conformity instead of genuine cooperative output. The result is that learners tend to regard collaboration as a performative requirement.

Compounding this is the emergence of what Bourdieu (1986) calls “misplaced investment in cultural capital.” In current policies such as the Recommended Admission to Graduate School system^4^, teamwork scores may contribute as little as 5% to overall assessment yet demand disproportionately high social and temporal costs. This imbalance incentivizes tactical compliance—such as superficial meetings, role-playing during teacher observation, or delegating most work to a single member.

The technological environment adds a further layer of complexity. Generation Z students, as Prensky describes “digital natives” (Kivunja, 2014), have developed collaboration norms in virtual spaces such as Genshin Impact^5^ and Honor of Kings, where skill-based trust is built through transparent, quantifiable performance metrics (e.g., DPS statistics). In contrast, classroom collaboration operates within a high-context cultural framework reliant on implicit cues and interpersonal negotiation. This high–low context mismatch (Hall, 1990) creates cognitive dissonance: students accustomed to low-context, data-driven collaboration struggle to adapt to the ambiguity of classroom assessments.

Moreover, the intensification of educational surveillance—through teacher monitoring, peer evaluation, classroom cameras, and digital learning platforms creates what Foucault terms a panoptic environment (Calderwood, 2025). Students respond with counter-technologies such as split-screen pseudo-participation and backchannel communication, engaging in what this study terms “strategic soloing” as a rational survival strategy. While these practices may ensure compliance, they undermine the intended benefits of PBL.

Existing research has documented PBL’s effectiveness in Western contexts but has largely overlooked how historical-cultural legacies, institutional evaluation regimes (Santhosh et al., 2023), and digital-age collaboration ethics intersect in non-Western educational settings. There remains limited understanding of how Chinese Generation Z students negotiate these competing influences in high-surveillance, high-stakes environments. Little research shows how collaboration mandates interact with students’ adaptive strategies, particularly when requirements run against motivation and efficiency.

This study addresses the following research questions:

RQ1. What makes strategic soloing a rational choice for Gen Z in PBL classrooms?
RQ2. How do formalism, institutional logics, and surveillance generate performative collaboration?
RQ3. How do virtual–real collaboration gaps shape students’ adaptive strategies?

This research situates PBL practices within the triple tension of imperial examination competition ethics, credit capitalism, and meta-cosmic cognition. Key concepts are defined in the study. Strategic soloing means that students deliberately reduce authentic cooperation and meet group requirements formally to improve efficiency, enhance control, and maximize personal credit. The imperial examination gene is used as an analytical construct, and it refers to historically rooted educational dispositions involving formalism, individual competition, and evaluation-oriented behaviors, all of which may shape contemporary practices. Credit capitalism is an institutional assessment system, in which academic activities become means of accumulating measurable credentials, thus collaboration becomes a cost–benefit decision. Meta-cosmic cognition refers to students’ familiarity with digital environments in which individual contributions are clearly recorded, ranked, and immediately visible. These concepts serve as analytical tools instead of empirical variables.

## 2 Literature review

The effectiveness of PBL has been widely verified in higher education around the world (Shin, 2018; (Zhang and Ma, 2023). Recent studies further show that PBL fosters learner autonomy, digital collaboration skills, and sustained engagement in technology-mediated classrooms (Mabe et al., 2022; Yiling et al., 2025). However, the practice of PBL in the context of Chinese higher education presents unique cultural tensions: on the one hand, collaborative learning has been shaped by policy texts as a key pathway for comprehensive quality cultivation (Eryong and Li, 2021); on the other hand, students’ tendency to strategically do it alone during the collaborative process exposes a deep-seated conflict between institutional design and socio-cultural genes. This conflict needs to be deconstructed from the multidimensional perspective of sociology of education and philosophy of technology.

The knowledge of PBL in existing research remains at the instrument rationality level, and Johnson and Mislin (2011) confirms in the meta-analysis that collaborative learning significantly improves academic achievement, but their findings based on the Western cultural context of individualism, fail to explain the phenomenon of isolation in collaboration among Chinese students. More recent cross-cultural research also emphasizes that collaborative outputs vary across sociocultural and institutional contexts, particularly in high-surveillance Chinese classrooms (Xu et al., 2024). Historically, the competitive ethic shaped by the imperial examination system continues to influence contemporary educational practices, with Elman (2013) noting that the Ming and Qing imperial examinations standardized knowledge through the system of vermilion ink transcription^6^, when students are more concerned with the process compliance of group work and are more concerned with mark scheme format than knowledge co-creation. This historical gene, combined with the synoptic calculus of Recommended Admission to Graduate School, has given rise to a unique logic of credit capitalism: the alienation of collaboration into a technical performance of “social capital accumulation” (Bourdieu, 1986).

The collaborative ethic of digital natives further exacerbates the institutional-behavioral disconnect, and Prensky’s notion of digital natives (Ponce Rojo et al., 2025) shows a culturally adapted variant in this study: the data-driven trust that Gen Z has developed in guilds of games, such as Genshin Impact that needs group cooperation creates a cognitive gap with the reality of classroom collaboration, which relies on the understanding of human sentiments. This fragmentation of real and virtual collaborative capabilities supports Don Ihde’s theory of technological embodiment, that is, meta-universal socialization reshapes students’ cognitive frameworks (Claassen, 2024), resulting in traditional classroom collaborative rules being reduced to inefficient systems. The intensification of educational surveillance technologies (e.g., classroom cameras, data tracking on SuperStar platform and WeChat observation) constitutes a panoramic open-view prison (Yiju, 2025), forcing students to struggle between performative collaboration and underground rebellion (e.g., counter-collaborative technologies such as split-screen playing and AI writing).

Existing studies have a triple limitation: first, they focus excessively on the operational techniques of PBL and ignore the dissolution of the meaning of collaboration by the institutional culture (e.g., the team score accounts only for 5% of the weighting in Recommended Admission to Graduate School); second, they fail to reveal the collaborative strategies of the digital natives in the interweaving of the virtual and the real; and third, they lack the dialectical analysis of the historical genes and technological impacts in the Chinese educational field. Through an ethnography approach, this study situates PBL practices in the triple tension of the ethics of imperial examination competition, credit capitalism and meta-cosmic cognition, providing a new theoretical lens for understanding the dilemmas of educational reform in Chinese higher education.

## 3 Methodology

This study employs an educational ethnography to reveal how Chinese Generation Z students navigate PBL in English classrooms. The approach integrates prolonged classroom observation, video-based micro-expression analysis, digital platform tracking, and in-depth interviews. It draws on Bourdieu’s field theory (Bourdieu, 1986), Foucault’s disciplinary power (Deacon, 2002), Elman’s historical lens (Elman, 2013), Ihde’s technological embodiment (Brey, 2000), and Prensky’s notion of digital natives (Kivunja, 2014), alongside more recent discussions of collaborative learning in high-surveillance environments (Higdon and Butler, 2024; Quiroz-Martinez and Rushton, 2024). Together, these frameworks illuminate the intersection of institutional structures, cultural legacies, and digital-era cognition in shaping collaboration.

### 3.1 Research design and philosophical stance

Situated in a qualitative, interpretivist paradigm, the study combines social constructivism, which foregrounds participants’ meaning-making within cultural-historical contexts (Mantula et al., 2024), and critical theory, which interrogates the power relations embedded in institutional and technological systems, is adopted. Ethnography was selected because it can capture tacit practices, symbolic codes, and resistance strategies *in situ*. The design assumes that collaborative behaviors emerge from both pedagogy and historically sedimented fields where imperial examination legacies and meta-universe collaboration ethics interact with contemporary evaluation regimes. The researcher serves as both observer and interpreter, examining how surveillance, credit capitalism, and game-derived norms inform student agency.

### 3.2 Research participants

The 150 first-year students in total from 4 classes (the classes of the researcher as well as the teacher) of one Chinese university were involved in physical classroom observation, video-recorded classroom observation, SuperStar platform observation, and WeChat group interaction and all belonged to the Generation Z cohort and identified as digital natives. Participants are aged between 17 and 20 years, and 108 male and 42 female students under the Automation Engineering program are involved. All of them were enrolled in compulsory College English courses and most come from lower-middle-class socioeconomic backgrounds (rural areas in China), consistent with the institutional profile of employment-oriented universities in China. None had prior PBL experience in English before being enrolled as a university student. Purposive sampling was adopted, and 20 interviewees were selected from 150 students based upon varied collaboration patterns (e.g., active contributors, passive members, and dominant solo performers), observable behavioral indicators of strategic soloing, and willingness to reflect, thus diverse perspectives and analytically meaningful insights were guaranteed. All participants had completed compulsory PBL units, experienced low-weight teamwork components in major assessments, and regularly engaged with digital platforms such as SuperStar and WeChat. They were approached as informants whose narratives and digital traces could reveal the cultural logic underpinning “strategic soloing.”

### 3.3 Data sources, collection, and analysis

Data collection spanned four months (September–December 2024) and drew on multiple complementary sources to capture interactional, digital, and perceptual dimensions of PBL group work. 40 h of classroom observation (CO1–CO40) across ten PBL units documented verbal and non-verbal behaviors, including resistance cues and performative collaboration under teacher proximity. All sessions were video-recorded (VCO1–VCO40) to enable micro-interaction analysis such as gaze shifts, eye-rolling, and split-screen device use. Video microanalysis followed a structured protocol. Each video was segmented into 10-min analytical units. Within each unit, observable behavioral indicators were coded, including gaze direction (screen, peers, teacher), verbal engagement frequency and duration, body orientation and posture changes, device interaction patterns (active editing, passive viewing, split-screen multitasking), and behavioral changes under teacher proximity. These indicators were linked to corresponding classroom observation notes and digital trace data, thus performative versus authentic collaboration can be analyzed comprehensively. Digital ethnography included extracting and analyzing SuperStar time-stamped logs (SS1–SS40), which revealed last-minute submission clusters and disproportionate labor contributions. WeChat group exchanges (WO, 1 September–31 December 2024) were monitored with informed consent to track task coordination, resistance language, and off-task communication. 20 semi-structured interviews (S1–S20) further explored students’ views on fairness, collaboration, and their comparison between gaming guilds and classroom groups. All interviews were audio-recorded, transcribed verbatim, and cross-referenced with observational and platform data.

The qualitative analysis followed Braun and Clarke’s (2022) reflexive thematic analysis and involved two coders. The first author acted as the primary coder, responsible for full-cycle coding, theme construction, and theoretical interpretation. The second coder—an experienced qualitative researcher—served as a reliability partner and critical auditor. Both coders independently open-coded 20% of the dataset (8 classroom observations, 4 interviews, 8 selected SuperStar logs, and WeChat exchanges) in NVivo 14. Their initial code sets were compared, discrepancies discussed (For example, in VCO12, a student performed active verbally when the teacher came close but immediately disengaged afterward. Initially, this behavior was coded as “situational participation” by coder 1 and “performative engagement” by coder 2. After discussion, both coders agreed to use “performative collaboration”), and a shared codebook with provisional definitions was established. Initial coding comparison revealed minor differences in approximately 12% of coded segments, primarily concerning distinctions between passive disengagement and strategic soloing. These discrepancies were resolved through repeated discussion and re-examination of video and log data. All disagreements were resolved rather than revolved after codebook refinement.

During the main coding phase, the author applied the codebook to the remaining data, inductively adding new codes where needed while documenting analytic decisions in memos. The second coder periodically reviewed coded extracts, theme maps, and memo trails, engaging in peer-debriefing meetings at three stages—early descriptive coding, mid-level clustering, and final theme refinement. Agreement was reached through negotiated interpretation rather than statistical indices; disagreements were resolved by returning to the raw data and reassessing contextual cues. Members checking with five student participants further ensured that preliminary interpretations were in line with their lived experiences. Theoretical saturation was reached after the 17th interview, since there were no emerging themes for coding. The remaining interviews and last 6 classroom observations consistently reinforced existing patterns and no new interpretive insights were generated afterword. Triangulation was achieved through a systematic cross-validation process. First, behavioral patterns in field classroom observation notes were matched with corresponding video units using synchronized timestamps. Second, digital trace data from the SuperStar platform were examined to verify actual contribution timing and frequency. Third, interview transcripts were analyzed to identify students’ subjective explanations for observed behaviors. For instance, when observation notes indicated students’ unwillingness to discuss in group meetings (CO18), video data confirmed it by recording passive body posture and limited verbal engagement (VCO18), while SuperStar logs showed single-user editing dominance (SS18), and interview data (S4) revealed students’ preference for individual task completion. This multi-source convergence enhanced interpretive validity. Discrepancies were resolved through iterative team discussions, thus credibility and validity were strengthened.

Each research question was addressed through targeted data–analysis alignment. RQ1, which explores why students prefer “strategic soloing,” relied primarily on classroom observations (CO1–CO40), interview transcripts (S1–S20), and SuperStar logs that revealed individual–group contribution imbalances. Comparative coding in NVivo identified recurrent patterns such as “instrumental cooperation,” “avoiding free riders,” and “efficiency-driven individualism.”

RQ2 examined how imperial-examination legacies, credit-oriented assessment mechanisms, and digital-native collaboration norms interact. This drew on triangulation across institutional documents (grading rubrics and assessment policies), video-recorded classroom observations (VCO1–VCO40), and interview narratives concerning fairness and workload distribution. From these, themes such as “imperial examination formalism” and “assessment-induced compliance” were iteratively refined.

RQ3, which investigates how surveillance practices and cross-space collaboration logics (classroom vs. gaming/guild cultures) shape adaptive strategies, utilized micro-interaction analysis of video data, WeChat group records (WO), and detailed interview accounts. Fine-grained coding captured gaze direction, speech-volume shifts, silent cooperation channels, and the emergence of “performative collaboration” and “underground coordination.” The integration of behavioral, linguistic, and perceptual evidence facilitated a cross-level explanation of students’ resistance under surveillance.

To clearly illustrate the data contributions to each research question and enhance methodological transparency, Table 1 provides an overview of the alignment between research questions, data sources, and analytic procedures. This table complements the narrative account above and clarifies the analytic architecture of the study.

**Table 1:** Overview of data sources and analytic focus by research questions.

Research question	Key data sources	Analytic focus
RQ1	Classroom observations (CO); Interviews (S); SuperStar logs (SS); Grading rubrics	Contribution imbalance; independent task control; evaluation-based effort allocation
RQ2	Institutional documents; Video-recorded observations (VCO); SuperStar logs; Interviews; WeChat records	Surveillance-triggered participation; performative collaboration; visibility-driven behavior shifts
RQ3	Video-recorded observations; SuperStar logs; WeChat records; Interviews	Digital–classroom contribution comparison; transparency expectations; strategic soloing adaptation

Where percentage data are reported in the findings and methodological descriptions, these figures represent different analytically grounded sources depending on context. Percentages such as “75% of interviewees” indicate the proportion of participants whose interview statements were coded under specific thematic categories, and this is consistent with qualitative research conventions. Percentages referring to grading structures (e.g., “30% of weight allocated to formatting” or “5% teamwork weighting”) are derived from institutional grading rubrics and policy documents. Methodological percentages (e.g., “20% of the dataset” or “12% coded segments”) imply procedural steps that ensure coding rigor and analytical transparency. Together, these percentage data serve descriptive and interpretive purposes within a qualitative ethnographic framework.

### 3.4 Research ethics

Ethical protocols followed institutional guidelines. Participants received detailed information sheets and signed consent forms covering classroom observation, digital monitoring, and interviews. Pseudonyms were assigned; facial images were blurred, and voiceprints modified to protect identities. WeChat monitoring was restricted to course-related groups, and participants could withdraw at any stage. Data was stored on encrypted devices, accessible only to the researcher and two independent auditors. Ethics approval was granted by the host institution, with special attention to managing the dual teacher-researcher role and potential power imbalances.

### 3.5 Reflection on researcher identity

The dual role of teacher and ethnographer provided insider access to “backstage” student discourse but risked reactivity effects. This dual role brought both advantages and risks. As instructor, the researcher had access to students’ daily conversations, SuperStar logs, WeChat interactions, and classroom behaviors, which would be difficult for external researchers to obtain, but this also risked authority-related response bias. Before the research began, classroom video recording was already a routine part of teaching in line with university regulation, and it was not something added specifically for the study. In addition, participants were told interviews were voluntary and unrelated to grade and assessment; and a second independent coder who has no teaching relationship challenged interpretations, thus minimizing insider bias.

Acknowledging that ethnography is inherently co-constructed (Hammersley, 2021), the researcher recognizes that presence may influence behaviors yet also affords unique insight into tacit negotiations within collaborative tasks. This reflexive awareness strengthens the credibility of the findings and situates them within the lived realities of both participants and researcher.

**Figure 1:**
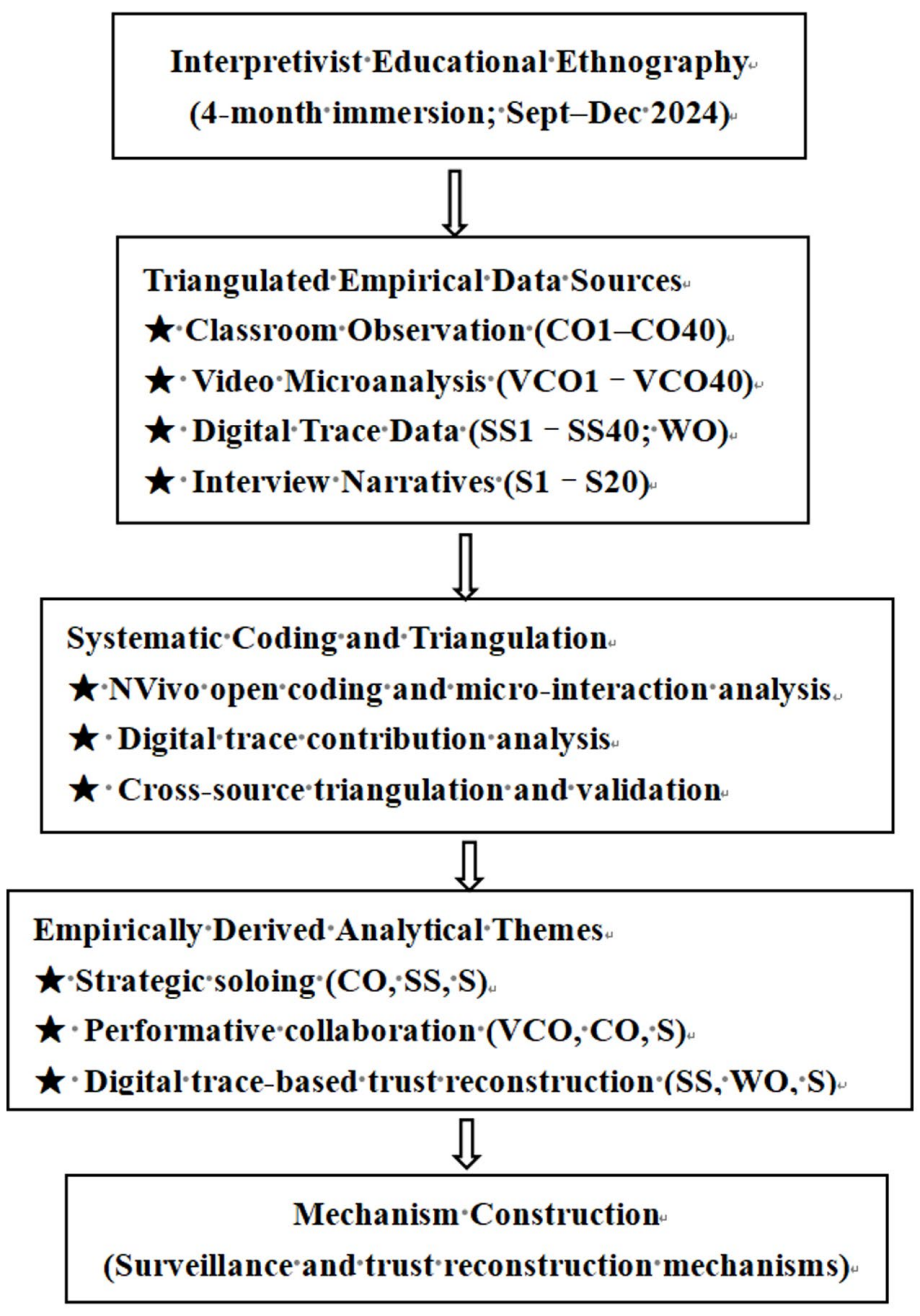
Research Methodology Framework.

The following figure represents the overall research methodological framework (Figure 1).

## 4 Results

The following sections show empirical findings based upon the research questions. Section 4.1 addresses RQ1 by demonstrating empirical evidence related to efficiency-driven soloing and assessment-based compliance. Section 4.2 addresses RQ2 by showing observation- and log-based evidence on surveillance and performative collaboration. Section 4.3 addresses RQ3 by demonstrating empirical evidence on cross-space collaboration comparisons and digital–classroom adaptation patterns.

### 4.1 Formalism-driven compliance and strategic soloing

#### 4.1.1 Formalism reproduction

The grading rubrics for group assignments gave as much as 30% of weight to document formatting (e.g., heading levels, font sizes), far outweighing the weight of creative realization (15%) and collaborative contribution (10%). This finding was triangulated through grading rubrics, classroom observations (CO12, CO18), and SuperStar logs (SS7, SS9), which showed prioritization of formatting over collaboration.

S1 said: “When the teacher checked the group report, the first thing he did was to turn the page to see if the page numbers are correct, just like the examiner of the imperial examination who counts the typos on the scrolls first. We spent 1 h on formatting but only 20 min discussing the content.” The researcher tracked assignment documents through the SuperStar platform (SS1–SS40), finding that over half the texts had signs of repeated formatting changes (on average, according to platform data, each document underwent 3.2 formatting adjustments), while content revision rarely occurred.

An interview with a Recommended Admission to Graduate School competitor (S15) reveals how institutional evaluation influenced behavioral priorities: “In the algorithm, the team score for group work only contributed to 5% of Recommended Admission to Graduate School points, but formatting errors will directly lead to loss of points. The rational choice, of course, is to prioritize preserving individual points, and collaboration is just a compliance performance.”

#### 4.1.2 Institutional incentive distortion

CO1–CO40 showed that students participated in offline meetings frequently to complete group work, but only two interviewees (S12 and S17) felt that these interactions contributed to language proficiency. This pattern was triangulated by SuperStar logs (e.g., SS19), revealing frequent meetings but uneven contributions and limited knowledge acquiring.

S3 explained this contradiction in gaming terms: “When you team up to play Genshin Impact, the DPS data determines everything; but when you team up to do an English presentation, it’s all about who has a good relationship with the teacher and who knows how to animate PPTs. Instead, it’s a ‘social gear competition’.” Observation data further documented performative participation patterns. In VCO21, when the teacher walked over to the group discussion area for the *Low Carbon Campus Initiative* project, students’ voice volume increased significantly and was accompanied by exaggerated body language such as nodding and pointing at the screen. When the teacher turned away, over half the groups quickly returned to silence or switched to WeChat private chatting.

#### 4.1.3 Virtual collaboration superiority and soloing behavior

Fifteen of 20 interviewees (75%) reported that collaboration in games such as Genshin Impact and Honor of Kings was more efficient than classroom cooperation. This perception was triangulated by WeChat records and classroom videos (VCO14, VCO27), showing limited authentic discussion and engagement.

In online battles, players built skill-based trust through real-time DPS statistics and contribution metrics (WO, 13 September 2024). In contrast, classroom group collaboration relied on vague contribution assessments, resulting in some interviewees (S8, S12, S14) experiencing free-rider dilemmas. S14 explained: “Gaming teams are ‘blockchain collaboration’ — everyone’s contribution is accurately recorded and cannot be tampered with. Teams are like a blind box, and you might open a box and find that your teammates have stuffed it with a bunch of junk.”

WeChat records showed that most discussions in course groups occurred within six hours before assignment deadlines and consisted primarily of procedural coordination such as “who will do the catalogue page” (WO, 19 October 2024).

S9 stated: “I can command a five-player group battle in Honor of Kings, but I’m afraid of offending people even when assigning tasks in an English group. Cooperation in authentic context is a hundred times harder than the game when you must consider ‘face credits’.”

SuperStar logs (SS3, SS8) and classroom observations (CO18–CO23) confirmed single-user editing dominance and individual device use during group assignments. S4 stated: “I think it is more efficient to complete tasks independently. I can control my own progress and quality without worrying about others dragging me backward.”

All these patterns indicate individual-centered task execution within formally collaborative structures.

To summarize the empirical patterns across rubric analysis, classroom observations, and platform records, Table 2 shows recurring correspondences between historical examination practices and contemporary assessment structures observed in this study.

**Table 2:** Empirical correspondences between historical examination practices and contemporary PBL assessment structures.

Historical practice	Contemporary assessment structure (observed)	Observed behavioral pattern
Vermilion ink transcription	Formatting weighted heavily in grading rubrics	Frequent formatting revisions in platform logs
Standardized examination essay templates	Fixed rubric-based report structure	Procedural compliance prioritized over content revision
Imperial examination competition logic	Low weighting of teamwork in evaluation	Uneven contribution and single-user task completion
Chengmo (procedural imitation strategies)	Teacher-monitored group discussion sessions	Increased visible participation under teacher observation

### 4.2 Surveillance and performative collaboration

#### 4.2.1 Surveillance control network

The combination of the Recommended Admission to Graduate School policy and monitoring technologies created a pervasive evaluation and monitoring environment within the educational field. Although teamwork scores were formally included as an indicator of students’ comprehensive evaluation, their weighting was limited to only 5% of the overall assessment.

As S9 stated: “The team score plays a very small part, but for that, I must spend so much time communicating and coordinating with others and sometimes dealing with interpersonal relationships. It’s really tiring.”

Surveillance operated through multiple channels, including direct teacher observation, classroom cameras, peer visibility, and digital platforms such as WeChat groups and the SuperStar learning system. This monitoring extended beyond physical classroom interaction into digital communication spaces.

S11 expressed this experience: “I feel like I am put in a transparent box, and I am watched by the teacher, as well as evaluated, and compared and watched by peers in everything I do. I even must be careful when I talk in the WeChat group.”

Observation records (CO13, CO15) documented behavioral adaptations such as split-screen device use, in which students displayed assignment-related content while simultaneously engaging in unrelated digital activities. WeChat records (WO, 1 September–31 December 2024) showed frequent asynchronous coordination occurring outside direct teacher observation.

SuperStar platform logs further confirmed that assignment activity often occurred outside observed classroom collaboration periods, indicating temporal separation between visible participation and actual task completion.

These observations, interview, and digital trace data all demonstrate that student collaboration occurred within a continuous multi-channel monitoring environment.

**Figure 2:**
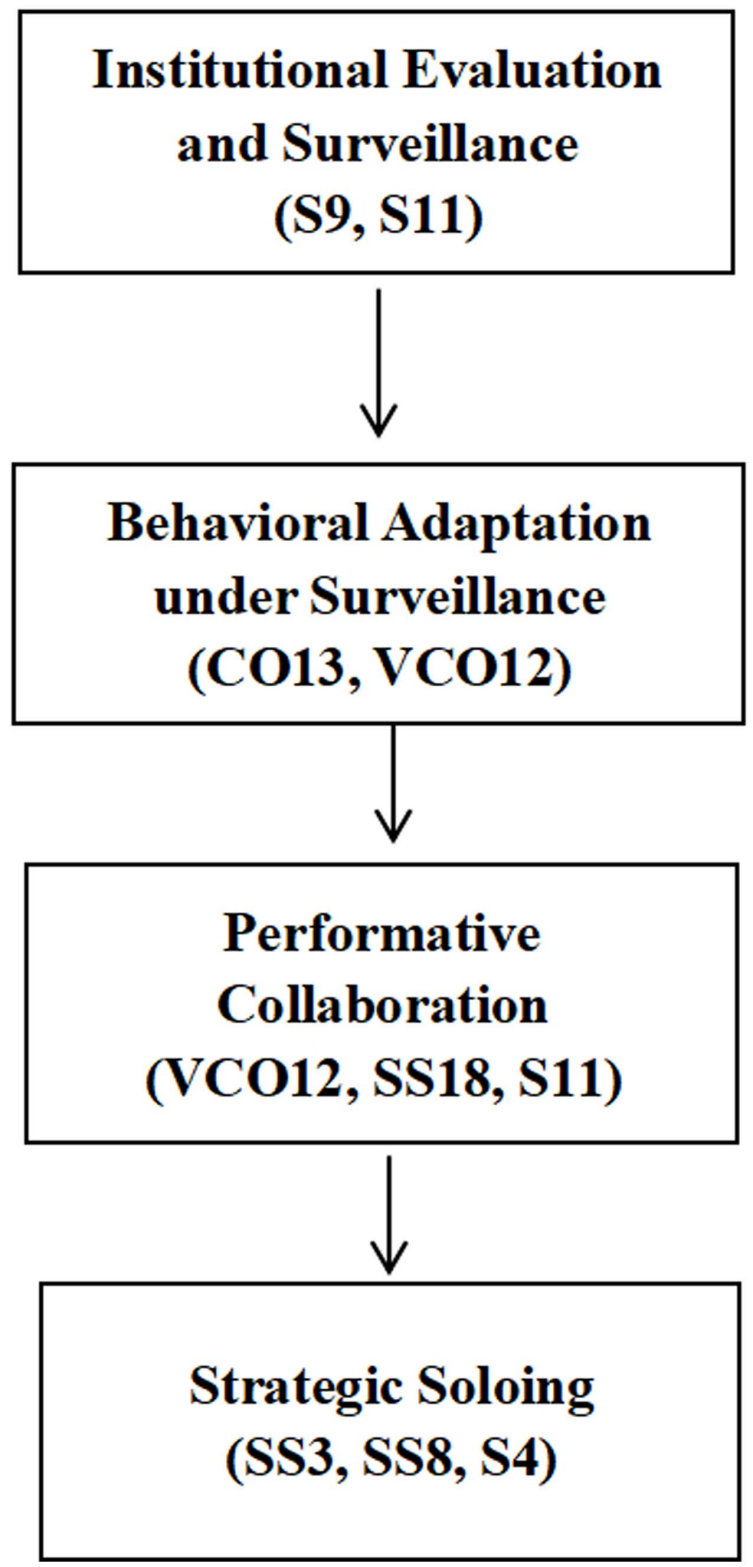
Empirical surveillance-mediated collaboration mechanism.

To integrate these empirical observations and clarify how institutional evaluation structures and surveillance visibility together shaped students’ collaborative behavior, Figure 2 demonstrates the empirically grounded mechanism, which links surveillance conditions to performative collaboration and strategic soloing.

#### 4.2.2 Performative collaboration behavior

Students gradually developed observable patterns of performative collaboration during classroom group activities. Video observation (VCO12) revealed that when the teacher came to the group discussion area for the *Campus Promotional Film* project, the voice volume of students’ discussion suddenly increased, accompanied by exaggerated body language and frequent head nodding. When the teacher turned around, the discussion quickly shifted to silence or was switched to WeChat private chat.

This behavioral pattern was triangulated by observation records, interview narratives, and digital trace data. Interviewees (e.g., S17, S11) described intentionally increasing visible participation when teachers were present, while SuperStar platform logs (e.g., SS18) showed minimal collaborative editing activity during these same observed discussion periods.

S17 stated: “We knew what the teacher was looking at, so we would pretend to be very active in front of the teacher, but we were not actually discussing the problem. “This was just to gain teacher’s good impression and hopefully get more marks in the general test.” Similarly, S11 reported that visible participation during observed group discussion did not always correspond to substantive task progress.

SuperStar platform records further showed that most document editing and substantive task completion occurred outside these observed classroom discussion periods.

These observations, interview, and platform log data consistently showed discrepancies between visible collaborative behavior and actual task execution during monitored classroom group work.

#### 4.2.3 Contribution imbalance and credit-oriented task completion

Data from the SuperStar platform and interviews (S12, S18, S19) showed that almost all group assignments tended to be completed primarily by a single person, while other group members had limited direct involvement in document editing and task execution.

SuperStar platform records further showed that discussions and coordination activities often occurred shortly before assignment deadlines, with communication concentrated within the final six hours prior to submission. These discussions primarily involved procedural coordination rather than substantive collaborative knowledge construction. This contribution imbalance was also observed directly in classroom interactions. CO9 and CO22 documented passive group members during task execution, while one student typically took primary responsibility for completing and submitting the assignment.

In the interviews, students expressed dissatisfaction with these collaboration patterns. S20 stated: “I think group work has become stale nowadays. We are not willing to really work together, and we wait until the last minute to start.” Similar patterns were reported by other interviewees (S12, S18, S19), who described completing assignments independently due to coordination difficulties and uncertainty regarding peer contributions.

Students’ concern over individual credit allocation could also be reflected. S8 said: “When contribution attribution was unclear, completing the assignment myself allowed me to maintain greater control over the grade.” In group settings where credit distribution was perceived as ambiguous or uneven, students expressed reluctance to rely on peers whose contributions could not be reliably verified (CO13-CO28). S8 added: “Independent task completion is an efficiency strategy and it’s also a mechanism for preserving my personal attributable academic credit.”

Uneven contribution and individual task control could be reflected in the triangulated evidence, and students’ strategic efforts to preserve credit certainty and minimize evaluation uncertainty were clear.

### 4.3 Digital-classroom collaboration conflict and adaptation

#### 4.3.1 High–low context collaboration conflict

Interview data confirmed that students relied on quantifiable indicators to assess collaboration effectiveness. S12 stated: “In Genshin Impact, I can clearly see the contribution of each teammate because DPS does not lie. But in classroom groups, I always think my efforts are ignored because the contributions are so vague.”

S14 stated: “Growing up in the digital age, I am accustomed to understanding and evaluating the world through data. However, when faced with the reality of classroom collaboration, I find myself having to put down the data labels and rely on vague contribution assessments and complex human relationships to judge the value of my teammates. This shift poses a huge challenge to me.” Similarly, S18 stated: “In the game, I can directly see the DPS of my teammates, but in the classroom group, I must look at who has a good relationship with the teacher and who can do PPT animation. I think it’s unfair.” Other interviewees also reported similar uncertainty regarding how contributions were recognized in classroom collaboration.

CO12-CO21 confirmed students’ rebellion in class: when arranged to complete the projects, project teammates had disputes with each other; after having disputes, most teammates kept silent and begun to play digital games despite the presence of teacher.

Interview records and classroom observations consistently showed that students perceived digital collaboration environments as providing clearer and more reliable contribution feedback compared to classroom collaboration contexts.

**Figure 3:**
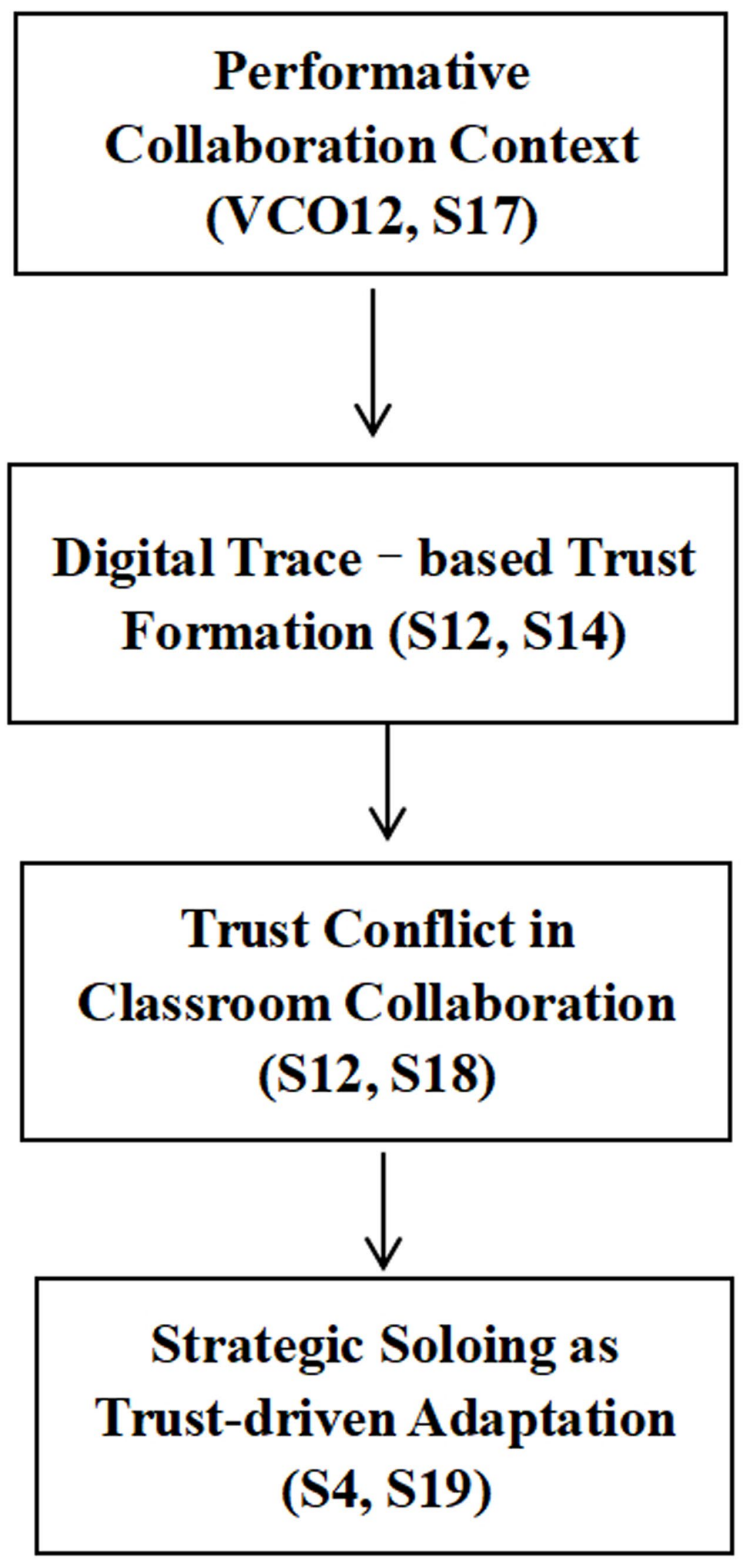
Trust reconstruction mechanism in digital and classroom collaboration.

To summarize the empirical findings, Figure 3 shows the trust reconstruction mechanism observed across digital and classroom collaboration contexts.

#### 4.3.2 Strategic soloing as behavioral adaptation

Observation records (e.g., CO18–CO23) showed frequent individual device use during group work, with limited direct collaborative interaction among group members.

SuperStar platform logs (e.g., SS3, SS8) further revealed single-user editing dominance, where one student typically assumed primary responsibility for completing and submitting the assignment, while other members had minimal direct editing involvement.

Interview data confirmed that students often preferred to complete tasks independently rather than collaboratively. This preference was reflected not only in behavioral records but also in students’ own descriptions of their task completion strategies. S4 explained: “I think it is more efficient to complete tasks independently. I can control my own progress and quality without worrying about others dragging me backward. I do not need to worry about peer evaluation and criticism. Also, there are a lot of online tools and resources available to help me complete tasks quickly.” Other interviewees (e.g., S19) reported similar experiences, indicating that individual task completion provided greater control over work quality, progress, and evaluation outcomes. Some interviewees (e.g., S3, S8) also questioned the necessity of collaboration in classroom group assignments, describing collaboration as a formal requirement rather than “an essential component of task completion.”

The triangulation of classroom observations, platform log data, and interview evidence consistently showed that strategic soloing functioned as a recurring behavioral pattern in classroom group assignments.

## 5 Discussion

This study explored why Generation Z students engaged in strategic soloing in project-based English classrooms and how institutional evaluation structures, surveillance environments, and digitally mediated collaboration experiences impacted this adaptive behavior. By triangulating classroom observations, interview data, and platform trace records, the study shows that strategic soloing does not mean disengagement from learning; instead, it is the restructuring of collaboration in specific institutional and technological conditions. Particularly, patterns including single-user editing dominance, surveillance-triggered participation shifts, and students’ preference for individual task completion show that collaboration was reorganized in response to perceived credit allocation asymmetry, visibility constraints, and contribution transparency expectations. This interpretation integrates psychological models with socioculturally situated evaluation logics, thus providing a framework for understanding how collaboration structures evolve under institutional and technological constraints.

### 5.1 Strategic soloing as adaptive credit regulation under asymmetric evaluation structures

The findings in Section 4.1 showed that students frequently prioritized formatting accuracy and independent task execution over collaboration. Platform logs recorded repeated formatting revisions alongside dominant single-user editing patterns, and interview participants preferred individual completion to avoid uncertainty related to uneven group contribution. These patterns show that strategic soloing reflected adaptive effort allocation rather than disengagement.

Expectancy–value theory provides a useful framework for interpreting this pattern, as learners regulate effort based on perceived effort–outcome contingencies (Wentzel and Miele, 2016). When evaluative credit is more directly associated with individually visible outputs than with collaborative contribution, students may prioritize activities that offer more predictable credit returns. In this study, institutional grading structures gave very limited weight to collaborative contribution relative to individually controllable performance indicators including formatting accuracy and submission completeness. On this basis, independently controlled task completion might reduce evaluation uncertainty and meanwhile enhance outcome predictability.

Similarly, research shows that students adjust their collaborative engagement in response to perceived evaluation transparency and accountability structures (Panadero et al., 2017). Therefore, the repeated procedural optimization observed in platform logs reveals adaptive credit regulation, in which learners invest effort selectively in activities, which are strongly linked to individual evaluation outcomes.

The concept of Chengmo^7^ (程墨), which is historically associated with procedural imitation strategies designed to optimize examination performance, provides a sociocultural lens for interpreting this pattern (Elman, 2013). Procedural optimization functions as an adaptive response to evaluation systems emphasizing formal compliance. In this study, strategic soloing demonstrates how learners reorganize effort allocation to be in line with institutional evaluation structures, which prioritize individually attributable performance.

From a self-regulated learning perspective, strategic soloing reveals adaptive effort regulation aimed at maximizing outcome controllability and minimizing evaluative risk (Panadero, 2017; Simón-Grábalos et al., 2025). Students remained engaged in task completion, but collaboration structures were selectively reorganized to preserve individual control over academic outcomes.

### 5.2 Surveillance visibility and counter-surveillance restructuring of collaboration

The findings in Section 4.2 showed that students’ observable collaboration behavior was sensitive to surveillance visibility. Video observations showed students’ increased participation when teachers approached groups, followed by their reduced interaction once observation ceased. Meanwhile platform logs showed that substantive document editing frequently occurred outside observed collaboration periods. This discrepancy suggests that collaboration was redistributed across different visibility contexts.

Self-determination theory shows that externally controlled environments can shift engagement toward externally regulated behavioral patterns (Ryan and Deci, 2020). Under continuous monitoring, students are likely to prioritize visible participation while maintaining internally regulated task execution strategies.

However, the present findings extend this interpretation by identifying counter-surveillance restructuring. Observation records documented split-screen multitasking, asynchronous coordination, and delayed task completion clustering. Meanwhile, platform logs confirmed that substantive editing was often completed by individual students outside direct observation. All these show that collaboration was reorganized spatially and temporally to balance institutional visibility expectations with individual outcome control.

Recent research supports this interpretation, suggesting that learners regulate both engagement intensity and engagement visibility under high-accountability environments (Johar et al., 2023). Thereby, visible participation may function as a compliance signal, while substantive task execution occurs through privately regulated processes.

The Chengmo concept again provides interpretive relevance, as procedural conformity historically served as a visible indicator of evaluation compliance. Similarly, performative collaboration in this study may serve as an observable signal of institutional alignment while allowing learners to maintain individual agency over task execution.

These findings extend self-regulated learning theory by showing that learners regulate not only effort intensity, but also the structural organization and visibility of collaboration under surveillance conditions.

### 5.3 Digitally embodied expectations of contribution transparency and collaboration cognition

The findings in Section 4.3 showed that students believed that digital collaboration environments provided clearer contribution transparency than classroom group work. Interview participants stressed the importance of visible contribution indicators and expressed uncertainty regarding how collaborative effort was evaluated in classroom settings.

Technological embodiment theory suggests that repeated interaction with digital systems shapes cognitive expectations regarding interaction and feedback structures (Brey, 2000). Digital collaboration environments provide continuous performance indicators, allowing participants to evaluate contribution objectively. In contrast, classroom collaboration often depends on implicit coordination, increasing uncertainty regarding contribution recognition.

Recent research shows that transparent contribution feedback strengthens collaboration trust and motivation (Wang et al., 2023; Wood and Pranjol, 2024). In this study, students reported difficulty in assigning tasks and evaluating peer contribution due to ambiguous evaluation standards and relational considerations. On this basis, independently controlled task completion may provide greater outcome predictability.

Importantly, these findings do not suggest that digital environments are inherently superior, but rather that digitally embodied expectations of contribution transparency shape how learners evaluate collaboration efficiency and fairness. Thereby, strategic soloing reflects adaptive adjustment to perceived mismatches between digitally embodied expectations and institutional evaluation structures.

### 5.4 Convergent and divergent evidence in collaboration research

The present findings both align with and diverge from existing research on collaborative learning, showing important boundary conditions for student participation in project-based environments. The observed tendency toward independently managed task completion is consistent with research suggesting that students regulate effort based on evaluation transparency and accountability. Expectancy–value theory and self-regulated learning research indicate that learners prioritize activities with predictable and individually attributable outcomes, particularly when collaborative contributions are difficult to verify (Panadero, 2017; Simón-Grábalos et al., 2025; Wentzel and Miele, 2016). In this regard, the strategic reorganization of collaboration in the study reveals students’ adaptive effort regulation under asymmetric evaluation conditions.

Meanwhile, these findings diverge from research showing that PBL and structured collaborative designs can enhance engagement and shared responsibility (Mohamadi, 2018; Sánchez-García and Reyes-de-Cózar, 2025). Prior research may suggest that transparent contribution evaluation and clear role allocation may strengthen participation and reduce free-riding. In contrast, the present study showed limited contribution visibility and asymmetric evaluation weighting reduced the perceived value of collaboration. This contrast shows that the patterns observed in this study do not necessarily reflect inherent limitations of collaborative learning, and instead it reveals the influence of specific institutional and evaluative conditions. Therefore, collaboration effectiveness depends on how evaluation structures and contribution visibility are implemented. Meanwhile, it shapes whether students engage in authentic cooperation or reorganize participation to preserve individual outcome predictability.

These findings further support the analytical lens proposed in this study, illustrating how collaboration restructuring emerges from the interaction between historically rooted examination ethics, institutional credit allocation structures, and digitally embodied expectations of contribution transparency.

### 5.5 Theoretical implications: strategic soloing as adaptive collaboration restructuring

This study contributes to psychological and educational theory by conceptualizing strategic soloing as an adaptive restructuring of collaboration emerging from the interaction between credit allocation asymmetry, surveillance visibility regulation, and digitally embodied expectations of contribution transparency. Strategic soloing reflects adaptive behavioral regulation aimed at maintaining outcome predictability and minimizing evaluation uncertainty in specific institutional conditions.

These findings empirically substantiate the proposed analytical framework integrating imperial examination competition ethics, credit capitalism, and meta-cosmic cognition, showing how historically sedimented evaluation logics, institutional credit structures, and digitally embodied contribution expectations shape adaptive collaboration restructuring together.

This interpretation extends self-regulated learning theory by showing that learners dynamically reorganize collaboration structures not merely individual effort in response to perceived evaluation contingencies. It also contributes to sociocultural psychology by showing how procedural optimization strategies, which are represented analytically through Chengmo, function as adaptive responses to formal evaluation environments.

Furthermore, the identification of counter-surveillance restructuring extends psychological models of engagement regulation by revealing how learners redistribute collaboration across visible and less visible interaction contexts to balance institutional requirements and individual outcome control.

Importantly, these findings are context-specific. The adaptive collaboration restructuring observed reflects the interaction between institutional evaluation structures, surveillance conditions, and digitally mediated collaboration experience in Chinese higher education context featuring asymmetric credit allocation, surveillance-mediated evaluation, and limited contribution transparency. Thereby, strategic soloing should be understood as a context-sensitive adaptive mechanism rather than a universal characteristic of collaborative learning.

Overall, collaboration behavior emerges as a dynamically regulated response to institutional credit structures, surveillance environments, and digitally mediated cognitive expectations. This framework provides a psychologically grounded explanation of how collaboration structures evolve under conditions of institutional and technological asymmetry.

## 6 Conclusion

This ethnographic study revealed how Generation Z students engaged in project-based English learning under mandatory collaboration requirements in the context of a Chinese employment-oriented university. Addressing RQ1, the findings reveal that strategic soloing functioned as an adaptive response to evaluation structures, which emphasize individually controllable outputs and limited recognition of collaborative contribution. Addressing RQ2, continuous monitoring through teachers, peers, and digital platforms shaped visible participation without strengthening substantive collaboration, leading students to regulate participation strategically. Addressing RQ3, digitally mediated collaboration experiences fostered expectations for transparent contribution tracking; when such transparency was absent, students preferred independently managed task execution. Overall, strategic soloing reveals adaptive collaboration restructuring in specific institutional and technological conditions instead of disengagement from learning.

At a theoretical level, this study contributes by empirically showing how collaboration in PBL may be reorganized as an adaptive behavioral response to specific institutional evaluation conditions, surveillance environments, and digitally shaped expectations of contribution transparency. Practically, these findings suggest that improving transparency in collaboration evaluation and aligning assessment structures with collaborative contribution may support more meaningful engagement in PBL. For example, incorporating clearer contribution tracking mechanisms, peer-verified task allocation, and assessment criteria that explicitly recognize collaborative processes may reduce uncertainty and enhance perceived fairness in group work. In addition, balancing monitoring practices with learner autonomy may help reduce performative participation and support authentic collaborative interaction.

This study has several limitations. First, it was conducted in a single institutional context only involving first-year students in one university, and collaboration practices may differ across institutional types, disciplines, and academic levels. Future research can examine diverse universities, disciplines, and student populations to explore contextual variation. Second, the focus on project-based English language may limit transferability to other disciplinary settings. Comparative studies across disciplines may provide broader insight. Third, the four-month ethnographic period may not capture longer-term adaptation processes; longitudinal research could examine how collaboration strategies evolve over time. Fourth, although multiple qualitative data sources were triangulated, future research may integrate mixed-method approaches, including quantitative behavioral tracking or social network analysis. Finally, the researcher’s dual role may have influenced participant behavior; independent or multi-site studies could further strengthen validity.

## Data Availability

The raw data (Chinese language) supporting the conclusions of this article are not publicly available at this time due to ethical, privacy, and data protection restrictions. Data may be made available by the author upon reasonable request, subject to institutional approval and compliance with participant confidentiality requirements. Requests to access the datasets should be directed to Guosheng Xu, Shandong Huayu University of Technology (1315329804@qq.com).

## Footnotes

1 A national higher education reform initiative integrating traditional humanities and social sciences with emerging technologies, interdisciplinary approaches, and practical innovation to meet the demands of the digital era and socio-economic transformation in China.

2 A civil service selection mechanism in imperial China (7th-20th century).

3 A Ming-Qing dynasty practice where imperial exam answers were copied in red ink by officials to ensure anonymous grading and prevent cheating.

4 A unique higher education pathway that allows top-performing undergraduate students to pursue master’s degrees without taking the national postgraduate entrance exams in China.

5 A free-to-play, open-world action RPG (role playing game) by Chinese developer.

6 An ancient formal assessment that has evolved into a cult of process compliance for group work in Contemporary China.

7 Official or exemplary essays compiled as standardized templates for candidates preparing China’s imperial examinations during the Ming and Qing dynasties.
